# *Curcuma longa* extract improves serum inflammatory markers and mental health in healthy participants who are overweight: a randomized, double-blind, placebo-controlled trial

**DOI:** 10.1186/s12937-021-00748-8

**Published:** 2021-11-13

**Authors:** Ryusei Uchio, Kengo Kawasaki, Chinatsu Okuda-Hanafusa, Ryosuke Saji, Koutarou Muroyama, Shinji Murosaki, Yoshihiro Yamamoto, Yoshitaka Hirose

**Affiliations:** Research & Development Institute, House Wellness Foods Corp., 3-20 Imoji, Itami, Hyogo 664-0011 Japan

**Keywords:** Turmeric (*Curcuma longa*), Bisacurone, Body mass index (BMI), Turmeronol, Chronic inflammation, C-reactive protein (CRP), Complement component 3 (C3), 36-item short-form health survey (SF-36), Profile of mood states (POMS)

## Abstract

**Background:**

The dietary spice *Curcuma longa*, also known as turmeric, has various biological effects. Both a water extract and a supercritical carbon dioxide extract of *C. longa* showed anti-inflammatory activities in animal studies. However, the anti-inflammatory effect in humans of a mixture of these two *C. longa* extracts (CLE) is poorly understood. Therefore, we investigated the effect of CLE containing anti-inflammatory turmeronols on chronic inflammation and general health.

**Methods:**

We performed a randomized, double-blind, placebo-controlled study in healthy subjects aged 50 to 69 years with overweight. Participants took two capsules containing CLE (CLE group, *n* = 45) or two placebo capsules (placebo group, n = 45) daily for 12 weeks, and serum inflammatory markers were measured. Participants also completed two questionnaires: the Medical Outcomes Study (MOS) 36-Item Short-Form Health Survey (SF-36) and the Profile of Mood States (POMS) scale. Treatment effects were analyzed by two way analysis of variance followed by a *t* test (significance level, *p* <  0.05).

**Results:**

After the intervention, the CLE group had a significantly lower body weight (p <  0.05) and body mass index (*p* < 0.05) than the placebo group and significantly lower serum levels of C-reactive protein (*p* < 0.05) and complement component 3 (*p* < 0.05). In addition, the CLE group showed significant improvement of the MOS SF-36 mental health score (*p* < 0.05) and POMS anger-hostility score (p < 0.05).

**Conclusion:**

CLE may ameliorate chronic low-grade inflammation and thus help to improve mental health and mood disturbance.

**Trial registration:**

UMIN-CTR, UMIN000037370. Registered 14 July 2019, https://upload.umin.ac.jp/cgi-open-bin/ctr/ctr_view.cgi?recptno=R000042607

**Supplementary Information:**

The online version contains supplementary material available at 10.1186/s12937-021-00748-8.

## Introduction

Inflammation is a physiological response to infection or tissue injury that involves vasodilatation, increased vascular permeability, and recruitment of leucocytes to inflammatory tissues to eliminate pathogenic microorganisms and dead cells, with subsequent induction of tissue repair and regeneration [1]. On the other hand, chronic inflammation is a persistent inflammatory response mediated by long-lived immune and non-immune cells that can have various undesirable effects, including tissue damage, inhibition of healing, promotion of fibrosis, and disruption of homeostasis [1, 2]. Recently, low-grade inflammation has been recognized as a state in which systemic inflammatory mediators are only slightly elevated relative to the levels seen in acute inflammation [3, 4]. This type of inflammation has not been clearly defined but it is considered to be related to aging [5], obesity [4], and an unhealthy lifestyle [6] in the absence of obvious infection or tissue injury [3]. Previous reports indicated that chronic low-grade inflammation is potentially associated with an increased risk of metabolic syndrome [7], atherosclerotic disease [8], Alzheimer’s disease [9], neurodegenerative disease [2], sickness behaviors and mood disturbance [10], cancer [11], and mortality [12, 13].

Inflammatory markers include C-reactive protein (CRP), complements, and fibrinogen, all of which are induced by inflammatory cytokines in response to stimulation by conserved microbial structures, tissue damage signals, and metabolic stress [1, 3, 4, 14]. CRP has been frequently used as a measure of low-grade inflammation and is well known to opsonize bacteria and apoptotic cells for their clearance via the complement systems and phagocytosis [15]. Additionally, CRP has been reported to promote pro-inflammatory cytokine/chemokine production in human macrophages [16] and to increase monocytes-endothelial cell adhesion [17]. Inflammation activates complement pathways, leading to the formation of two different complement component 3 (C3) convertases: C4bC2b in the classical and lectin pathways and C3bBb in the alternative pathway. These C3 convertases cleave C3 into two fragments: C3a, an inflammatory mediator, and C3b, which induces the formation of lytic membrane attack complexes for lysing targeted cells and bacteria [18]. Fibrinogen has an important role not only in the activation of the coagulation cascade but also in the promotion of inflammation by inducing cytokine production, leukocyte infiltration in inflamed tissues, and platelet aggregation [19]. High systemic levels of these inflammatory markers are well known to be associated with increased risk for chronic inflammatory diseases [2, 19, 20]. In addition, elevation of circulating levels of CRP and C3 may be associated with impaired quality of life (QOL) and mood disturbance [21–23].

*Curcuma longa*, also known as turmeric, is a member of the Zingiberaceae family and a traditional spice that has various physiological activities [24]. Water extracts of turmeric have antioxidant and anti-inflammatory effects [25], promote corneal wound healing [26], show antidepressant activity [27], and have an anticancer effect [28]. In addition, hot water extracts of *C. longa* were reported to prevent various chronic inflammatory diseases in animal models, including cotton pellet-induced granuloma [29], carbon tetrachloride-induced hepatitis [30], and non-alcoholic steatohepatitis [31, 32], by decreasing the expression of genes coding for inflammatory cytokines and cell adhesion molecules. Supercritical carbon dioxide extracts of *C. longa* also were shown to have antioxidant activity in vitro and to inhibit carrageenan-induced inflammation in the rat paw [33]. However, the influence of a mixture of a hot water extract and a supercritical carbon dioxide extract of *C. longa* on chronic inflammation and general health conditions in humans is not clearly understood.

To evaluate the effect of a mixture of both *C. longa* extracts on chronic inflammation and associated health conditions, we measured blood levels of inflammatory markers and assessed general health with two questionnaires, the Medical Outcomes Study (MOS) 36-Item Short-Form Health Survey (SF-36) and the Profile of Mood States (POMS) scale, in middle-aged and elderly participants with overweight.

## Materials and methods

### Study design

**Fig. 1:**
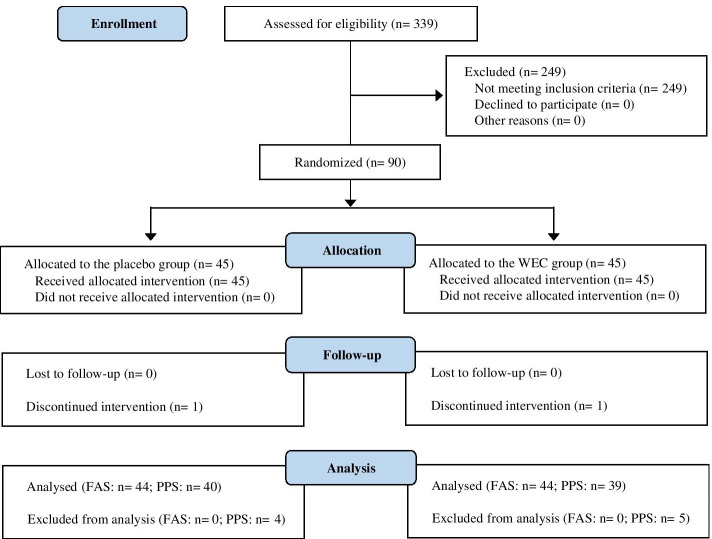
Study flow diagram (CONSORT 2010)

This was a 12-week, randomized, double-blind, placebo-controlled interventional study. All procedures involving human participants were approved by the institutional review board of Chiyoda Paramedical Care Clinic (Tokyo, Japan), and the study was conducted according to the Declaration of Helsinki. Before enrollment in the study, written informed consent was obtained from all participants. This study was performed by a contract research organization (CRO; CPCC Co., Ltd., Tokyo, Japan) at the Chiyoda Paramedical Care Clinic from June to December 2019 and was registered with the University hospital Medical Information Network (UMIN; Registration number, UMIN000037370). Figure 1 shows the Consolidated Standards of Reporting Trials (CONSORT) 2010 diagram of the flow of participants from enrollment to analysis [34]; the completed CONSORT checklist is provided in Table S1.

### Enrollment of participants

Participants were recruited from July to September 2019. A total of 339 potential participants among people attending the Chiyoda Paramedical Care Clinic were consecutively assessed for eligibility. The inclusion criteria were age 50 to 69 years, overweight (body mass index [BMI] ≥ 23 to < 30 kg/m^2^) [35], blood glucose below 126 mg/dL, and blood high-density cholesterol greater than or equal to 35 mg/dL; both men and menopausal women were included. The exclusion criteria were as follows: (1) positive test for hepatitis C virus antibody or hepatitis B surface antigen, (2) use of medications or health foods that could possibly influence the results of this study, (3) history of heart, liver, kidney, or gastrointestinal disease, (4) history of circulatory disease, (5) excessive alcohol intake (mean daily consumption of 60 g or more), (6) excessive smoking (mean daily consumption of two packs or more), (7) extremely irregular dietary habits, (8) allergies to medications or foods (especially soybeans and gelatin), (9) participation in another trial, either currently or in the past 4 weeks, or plan to participate in another trial during the scheduled study period, (10) donation of blood within 1 month before the study, (11) males who have made a blood donation of 400 mL of blood within 3 months before the study, (12) females who made a blood donation of 400 mL within 4 months before the study, (13) males who have made a blood donation over an amount (1200 mL minus the estimated volume of blood collected during the study within 1 year before the study, (14) females who have made a blood donation over an amount (800 mL minus the estimated volume of blood collected during the study) within 1 year before the study, (15) judged to be unsuitable for the study for other reasons by the investigators.

### Study agent

The composition of the capsules used in this study is described in Table 1. The base capsules were composed of gelatin, glycerin, soybean-derived emulsifier, and beeswax. Placebo capsules also contained carob and tartrazine as coloring agents so that they matched the color of the turmeric capsules, and turmeric capsules contained a mixture of a hot water and a supercritical carbon dioxide extract of *C. longa* (CLE, Turmeric Extract Mixture, House Wellness Foods). The extracts of *C. longa* were prepared according to the method described previously [36–38]. In brief, to obtain the hot water extract of *C. longa* powder, the rhizomes of *C. longa* were extracted for 1 h with hot water at 98 °C, after which the supernatant was concentrated, mixed with dextrin, and spray dried. To obtain a supercritical carbon dioxide extract of *C. longa* oil, the rhizomes of *C. longa* were extracted for 6 h with carbon dioxide at 65 °C and 150 bar, after which the supernatant was concentrated, mixed with vegetable oil, and filtered through a membrane filter (1-μm pore size).

**Table 1:** Composition of the study capsules

	Placebo (0.97 g/2 capsules)	CLE (0.97 g/2 capsules)
Energy, Kcal	6.1	5.5
Carbohydrate, g	0.24	0.23
Protein, g	0.20	0.29
Lipid, g	0.47	0.38
Sodium chloride, mg	0.66	0.13
Bisacurone, μg	0	400
Turmeronol A, μg	0	100
Turmeronol B, μg	0	100

CLE: a mixture of a hot water extract and a supercritical carbon dioxide extract of *Curcuma longa*

### Intervention

The intervention was conducted from September to December 2019. From the 339 potential participants, the CRO selected 90 participants who satisfied the inclusion and exclusion criteria, had no symptoms associated with acute inflammation, as assessed by their physicians. The test capsules and selected participants were randomly assigned numbers by the authors and the CRO, respectively, and the randomization list was stored carefully until the database was locked. Throughout the study, all participants and investigators were blinded to the treatment group. The participants were randomly allocated to the two groups by stratified randomization on the basis of sex, age, BMI, and CRP level. Each participant took two capsules containing CLE (CLE group, *n* = 45) or two placebo capsules (placebo group, n = 45) once daily for 12 weeks. Participants visited the study center in weeks 0, 4, 8, and 12 to undergo an interview by an experienced physician, physical measurements and tests, hematology and biochemistry tests, and urinalysis and to complete the questionnaires; adverse events were assessed at each visit. Hematology and biochemistry tests and urinalysis were performed by a contract laboratory company (LSI Medience Co., Ltd., Tokyo, Japan), and the questionnaires were evaluated by the CRO. The participants were asked to record the following information on a daily basis for the duration of the study: occurrence of diseases and symptoms; intake of study capsules, health foods, and medications; and reasons for taking medications, dosages, and duration of use. They were also asked to adhere to the following rules until the end of the study: (1) maintain their usual lifestyle through the study period, including diet, exercise, drinking, smoking, and medication; (2) avoid consuming health foods other than the study capsules; (3) avoid donating blood; and (4) avoid excessive drinking, smoking, exercise, fasting, and consumption of an unbalanced diet. Participants were instructed to fast, except for drinking water, after 9:00 pm on the day before each examination.

### Measurement of body weight and BMI

Body weight was measured using a calibrated digital scale. BMI was calculated as body weight in kilograms divided by height in meters squared (kg/m^2^).

### Measurement of inflammatory markers

The serum level of high-sensitivity CRP (hsCRP) was measured by an immunonephelometric method with an upper detection limit of 0.500 mg/dL. For samples exceeding this limit, a remeasurement was performed by standard latex agglutination turbidimetry. The serum levels of C3 and C4 were determined by turbidimetric immunoassays, and plasma fibrinogen levels were measured with a thrombin method.

### The medical outcomes study 36-item short-form health survey

The second edition of the MOS 36-Item Short-Form Health Survey (SF-36) is a widely used self-reported questionnaire that evaluates health-related QOL and has demonstrated good reliability and validity [39, 40]. Items are aggregated into the following eight subscales: physical functioning (PF), role physical (RP), bodily pain (BP), general health (GH), vitality (VT), social functioning (SF), role emotional (RE), and mental health (MH). In our study, all items were transformed and summed according to the official manual to give scores from 0 to 100 points for each subscale, with higher scores indicating better QOL. The scores for the eight subscales were then used to obtain scores for the two main components, ie, the physical component summary (PCS) score and the mental component summary (MCS) score. Three subscales (PF, RP, and BP) are the main determinants of the PCS; three others (SF, RE, and MH) are the main determinants of the MCS. The remaining two scales (GH and VT) were included in the calculation of both component summary scores. Then, the sums of the PCS and MCS were each multiplied by 10, and 50 was added to achieve linear transformation to the T-score metric, which has a mean of 50 and a standard deviation of 10 in the general Japanese population.

### Profile of mood states scale

The second edition of the Profile of Mood States (POMS) scale short version is a widely used self-reported questionnaire for rapidly assessing mood states, including both transient, fluctuating feelings and enduring affect states [41, 42]. The POMS scale contains 35 questions that assess the following seven moods: anger-hostility (AH), confusion-bewilderment (CB), depression-dejection (DD), fatigue-inertia (FI), tension-anxiety (TA), vigor-activity (VA), and friendliness (F). Participants were asked to indicate their mood states during the previous week on a 5-point scale ranging from “not-at-all” to “extremely.” Total mood disturbance (TMD) scores were calculated for each subscale by the following formula: TMD = AH + CB + DD + FI + TA - VA. High scores for VA and F indicate a positive mood state, whereas high scores for AH, CB, DD, FI, and TA and a high TMD indicate a negative mood state.

### Sample size

To calculate the minimum number of participants required for adequate statistical power, we used the G Power 3.1.9 program (University of Dusseldorf, Germany). In a previous clinical study, a green tea extract with anti-inflammatory activity reduced the serum CRP level by about 30% from baseline [43]. Therefore, a sample size of 45 participants per group was estimated to be sufficient for the present study on the basis of the following assumptions: 30% reduction of serum CRP by CLE, Cohen’s d value = 0.60, statistical power = 80%, and type I error = 5% (two-tailed).

### Statistical analysis

Statistical analysis was performed in the intention-to-treat (ITT) population, which was defined as all randomized participants. The full analysis set (FAS) was used to assess safety, and the per-protocol set (PPS) was used to evaluate efficacy. In the efficacy assessment of inflammatory markers, we excluded the data of participants who were suspected to have acute inflammation because of a markedly increasing CRP or fibrinogen value or symptoms associated with acute inflammation, as diagnosed by a physician (common cold symptoms, acute lower back pain, physical injury, or proteinuria). All statistical analyses were performed with the IBM SPSS statistical software package (version 26) for Windows (IBM Corp., New York, USA). Results are presented as the mean (standard deviation [SD]). Baseline characteristics were compared between the two groups by the two-tailed unpaired Student’s *t* test when variance was homogeneous or the Aspin-Welch *t* test when variance was heterogeneous, except for the results of sex and urinalysis, which were analyzed by the two-tailed Mann Whitney *U* test. Changes from baseline were analyzed by repeated measures two-way analysis of variance (ANOVA; two groups × three time points) with the SPSS general linear model for determining the main effects of group and time and their interaction, followed by comparison between the placebo and CLE groups at each time point with the two-tailed unpaired Student’s *t* test when variance was homogeneous or the Aspin-Welch *t* test when variance was heterogeneous. A probability (p) value less than 0.05 was considered to indicate statistical significance.

## Results

### Participants

The flow of participants through the trial is shown in Fig. 1. A total of 90 out of 339 potential participants were randomly allocated to the CLE group or the placebo group (*n* = 45 per group). Two participants dropped out before completing the study: One was in the placebo group and discontinued the study because of a marked reduction of body weight over the course of the study, and one was in the CLE group and declined to continue to participate because of a bone fracture resulting from a slip-and-fall accident while walking. Nine participants were excluded from the efficacy assessment (PPS analysis) because they did not comply with the protocol for the following reasons: change of dietary habits (*n* = 4 in the CLE group and n = 4 in the placebo group), and bone fracture resulting from a slip-and-fall accident while cycling (*n* = 1 in the CLE group). The other 79 participants completed the study. Baseline characteristics showed no significant differences between the CLE and placebo groups, except for a significantly higher serum C4 and glucose levels in the placebo group than in the CLE group (Tables 2 and 3). Both groups met the requirements for the mean [SD] intake of capsules (CLE group, 100.0% [0.2%]; placebo group, 99.8% [0.6%]). Exercise was performed during the study by 11 participants in the CLE group and 12 in the placebo group and was not performed 28 participants in each group; the number of participants who exercised was not significantly different between the two groups (*p* = 0.862). In addition, among the participants who exercised, the mean [SD] number of days of exercise was not significantly different between the two groups (CLE group, 4.3 [9.1] days; placebo group, 4.0 [8.6] days; *p* = 0.868).

**Table 2:** Baseline characteristics of the participants^a^

	Placebo group (*n* = 40)		CLE group (*n* = 39)		*p* value
	Mean	SD	Mean	SD	
Sex, male/female, n	16/24		15/24		0.889
Age, years	56.6	4.3	56.7	5.0	0.930
Physical measurements and tests					
Height, cm	162.5	8.5	162.5	8.5	0.985
Body weight, kg	69.9	8.0	70.3	7.8	0.821
BMI, kg/m^2^	26.4	1.8	26.6	1.5	0.664
SBP, mmHg	122.9	12.8	119.7	14.7	0.308
DBP, mmHg	77.6	10.2	77.6	9.6	0.995
Serum inflammatory markers					
CRP, mg/dL	0.076	0.047	0.075	0.064	0.940
C3, mg/dL	110.8	12.6	104.7	14.2	0.073
C4, mg/dL	27.5	7.0	24.0	6.0	0.037
Fibrinogen, mg/dL	285.7	43.8	288.5	45.0	0.797
Metabolic markers					
Glucose, mg/dL	92.3	8.7	88.5	6.4	0.031
HbA1c, %	5.63	0.20	5.55	0.26	0.147
Triglyceride, mg/dL	120.8	52.9	122.2	67.0	0.915
Total cholesterol, mg/dL	231.4	36.8	221.2	37.5	0.226
LDL-cholesterol, mg/dL	148.7	31.9	138.2	31.9	0.151
HDL-cholesterol, mg/dL	58.2	11.9	58.2	16.6	0.980

*BMI* body mass index, *CLE* a mixture of a hot water extract and a supercritical carbon dioxide extract of *Curcuma longa*, *DBP* diastolic blood pressure, *HbA1c* hemoglobin A1c, *HDL* high-density lipoprotein, *CRP* C-reactive protein, *C3* complement component 3, *C4* complement component 4, *LDL* low-density lipoprotein

^a^Values represent the means and standard deviations for n = 40 (placebo group) or n = 39 (CLE group). Comparison of sex was performed with the two-tailed Mann Whitney *U* test. Comparisons of physical measurements and tests, serum inflammatory markers, and metabolic markers were performed with the two-tailed unpaired Student’s *t* test when variance was homogeneous or the Aspin-Welch *t* test when variance was heterogeneous

**Table 3:** Baseline scores for the MOS 36-Item Short-Form Health Survey (SF-36) and Profile of Mood States (POMS) questionnaire^a^

	Placebo group (*n* = 40)		CLE group (*n* = 39)		*p* value
	Mean	SD	Mean	SD	
SF-36 scores, points					
Physical functioning (PF)	92.5	6.8	91.8	12.8	0.762
Role physical (RP)	94.7	10.2	93.1	13.4	0.558
Bodily pain (BP)	88.3	13.5	89.0	16.6	0.826
General health (GH)	71.7	13.9	77.6	13.9	0.060
Vitality (VT)	65.3	16.1	69.9	20.4	0.269
Social functioning (SF)	94.7	10.2	91.7	14.4	0.287
Role emotional (RE)	94.4	10.9	92.9	13.2	0.599
Mental health (MH)	80.1	12.2	79.4	15.4	0.812
Physical component summary (PCS)	53.4	4.4	52.2	7.0	0.382
Mental component summary (MCS)	56.5	5.9	58.0	8.8	0.358
POMS scores, points					
Anger-hostility (AH)	45.5	6.9	45.4	6.2	0.979
Confusion-bewilderment (CB)	46.9	6.9	46.0	8.1	0.619
Depression-dejection (DD)	47.3	6.8	47.0	7.5	0.877
Fatigue-inertia (FI)	45.2	5.8	44.2	8.1	0.540
Tension-anxiety (TA)	48.7	8.9	45.9	7.8	0.142
Vigor-activity (VA)	54.4	9.1	56.3	9.2	0.362
Friendliness (F)	56.3	9.4	56.5	9.3	0.901
Total mood disturbance (TMD)	45.3	6.8	43.7	8.0	0.332

CLE: a mixture of a hot water extract and a supercritical carbon dioxide extract of *Curcuma longa*

^a^Values represent the means and standard deviations for n = 40 (placebo group) or n = 39 (CLE group). Comparisons of SF-36 and POMS data were performed with the two-tailed unpaired Student’s *t* test when variance was homogeneous or the Aspin-Welch *t* test when variance was heterogeneous

### Effect of CLE on body weight and BMI

The change of body weight from baseline was significantly lower in the CLE group than in the placebo group throughout the whole study period (*p* = 0.009 by repeated measures two-way ANOVA [r-ANOVA]) and they were significantly lower in weeks 4, 8, and 12 in the CLE group compared with the placebo group (Table 4) Similarly, the change of BMI from baseline was significantly lower in the CLE group than in the placebo group throughout the whole study period (*p* = 0.007 by r-ANOVA) and they were significantly lower in weeks 4, 8, and 12 in the CLE group compared with the placebo group (Table 4).

**Table 4:** Effect of *Curcuma longa* extract (CLE) on body weight and body mass index^a^

	Change from baseline						Repeated measures 2-way ANOVA		
	Week 4		Week 8		Week 12		Group	Time	Interaction
	Mean	SD	Mean	SD	Mean	SD			
Body weight, kg									
Placebo	0.31	0.72	0.52	0.93	0.65	0.86	0.009	0.037	0.583
CLE	−0.18*	1.08	−0.04*	1.18	−0.03*	1.49			
BMI, kg/m^2^									
Placebo	0.12	0.28	0.20	0.38	0.26	0.33	0.007	0.023	0.520
CLE	−0.08*	0.41	−0.02*	0.46	−0.02*	0.57			

*BMI* body mass index

^a^Values represent the means and standard deviations for n = 40 (placebo group) or n = 39 (CLE group). *p < 0.05: Significant difference from the placebo group assessed by repeated measures two-way ANOVA, followed by the two-tailed unpaired Student’s *t* test when variance was homogeneous or the Aspin-Welch *t* test when variance was heterogeneous

### Effect of CLE on inflammatory markers

The change of CRP from baseline was tended to be lower in the CLE group than in the placebo group throughout the whole study period (*p* = 0.057 by r-ANOVA) and it was tended to be lower in week 8 (*p* = 0.073) and significantly lower in week 12 (*p* < 0.05) in the CLE group compared with the placebo group (Table 5). The changes of C3 from baseline showed no significant differences between the two groups throughout the whole study period (r-ANOVA), but it was significantly lower in week 12 in the CLE group than in the placebo group (Table 5). The changes of C4 and fibrinogen did not show significant differences between the two groups.

**Table 5:** Effect of *Curcuma longa* extract (CLE) on serum inflammatory markers^a^

	Change from baseline						Repeated measures two-way ANOVA		
	Week 4		Week 8		Week 12		Group	Time	Interaction
	Mean	SD	Mean	SD	Mean	SD			
CRP, mg/dL									
Placebo	0.019	0.044	0.036	0.091	0.016	0.047	0.057	0.363	0.302
CLE	0.021	0.089	0.002	0.032	−0.007*	0.035			
C3, mg/dL									
Placebo	4.13	7.94	0.10	9.33	7.26	9.00	0.514	< 0.001	0.138
CLE	4.00	8.14	1.41	11.90	2.66*	8.20			
C4, mg/dL									
Placebo	0.06	2.78	0.71	3.57	1.48	3.47	0.366	0.094	0.135
CLE	0.28	2.58	−0.07	3.42	0.34	2.69			
Fibrinogen, mg/dL									
Placebo	9.0	27.9	11.4	46.3	15.1	35.5	0.648	0.371	0.950
CLE	5.3	50.6	3.7	29.7	12.0	37.8			

CRP: C-reactive protein; C3: complement component 3; C4: complement component 4

^a^Values represent the means and standard deviations at 4, 8, and 12 weeks for *n* = 31, 31, and 31 (placebo group) or *n* = 29, 27, and 29 (CLE group), respectively. *p < 0.05: Significant difference from the placebo group by repeated measures two-way ANOVA, followed by the two-tailed unpaired Student’s *t* test when variance was homogeneous or the Aspin-Welch *t* test when variance was heterogeneous

### Effect of CLE on SF-36 scores

The changes of MH scores from baseline were significantly higher in the CLE group than in the placebo group over the whole study period (*p* = 0.032, r-ANOVA) and it was significantly higher in week 8 in the CLE group compared with the placebo group (Table 6). The scores for the remaining seven SF-36 subscales did not show significant differences between the two groups.

**Table 6:** Effect of *Curcuma longa* extract (CLE) on the MOS 36-Item Short-Form Health Survey (SF-36) scores^a^

	Change from baseline						Repeated measures two-way ANOVA		
	Week 4		Week 8		Week 12		Group	Time	Interaction
	Mean	SD	Mean	SD	Mean	SD			
Physical functioning (PF)									
Placebo	1.00	7.18	0.38	7.20	0.25	6.20	0.768	0.081	0.343
CLE	0.77	5.91	1.71	5.61	0.64	7.18			
Role physical (RP)									
Placebo	1.25	10.60	0.78	12.10	1.87	8.27	0.719	0.158	0.538
CLE	−0.65	12.40	0.16	9.36	2.24	10.08			
Bodily pain (BP)									
Placebo	−0.8	14.0	−2.0	14.3	−2.1	11.4	0.544	0.612	0.999
CLE	−2.4	14.9	−3.1	13.9	−3.7	15.6			
General health (GH)									
Placebo	−0.30	10.99	0.18	13.35	0.63	11.71	0.121	0.881	0.884
CLE	−3.08	9.73	−3.58	10.91	−3.05	12.80			
Vitality (VT)									
Placebo	−2.51	14.36	−0.94	11.88	1.71	10.49	0.692	0.040	0.734
CLE	−2.56	13.28	−1.32	11.99	−0.32	11.99			
Social functioning (SF)									
Placebo	−0.94	13.69	0.31	14.57	−0.63	13.56	0.438	0.221	0.749
CLE	0.32	18.02	3.95	14.85	1.92	12.35			
Role emotional (RE)									
Placebo	0.63	11.99	0.84	12.20	1.04	12.27	0.924	0.648	0.850
CLE	−0.21	13.04	0.88	11.76	1.49	11.78			
Mental health (MH)									
Placebo	−3.63	10.50	−3.38	11.29	−1.75	9.37	0.032	0.127	0.416
CLE	−0.90	9.86	2.89*	10.24	1.92	10.68			
Physical component summary (PCS)									
Placebo	1.22	4.85	0.77	4.66	0.54	3.67	0.574	0.836	0.491
CLE	0.23	4.97	0.28	4.02	0.52	4.63			
Mental component summary (MCS)									
Placebo	−1.76	5.10	−1.18	5.33	−0.38	4.39	0.618	0.144	0.766
CLE	−1.21	5.72	−0.10	4.55	−0.37	5.24			

^a^Values represent the means and standard deviations for n = 40 (placebo group) or n = 39 (CLE group). *p < 0.05: Significant difference from the placebo group by repeated measures two-way ANOVA, followed by the two-tailed unpaired Student’s *t* test when variance was homogeneous or the Aspin-Welch *t* test when variance was heterogeneous

### Effect of CLE on POMS scores

The change of the AH score from baseline was significantly lower in CLE group than the placebo group throughout the study period (*p* = 0.022, r-ANOVA) and it was significantly lower in weeks 4 and 12 in the CLE group compared with the placebo group (Table 7). The other POMS scores did not show significant differences between the two groups.

**Table 7:** Effect of *Curcuma longa* extract (CLE) on the Profile of Mood States (POMS) scores^a^

	Change from baseline						Repeated measures two-way ANOVA		
	Week 4		Week 8		Week 12		Group	Time	Interaction
	Mean	SD	Mean	SD	Mean	SD			
Anger-hostility (AH)									
Placebo	1.95	4.76	1.55	6.02	1.75	5.29	0.022	0.706	0.472
CLE	−0.69*	5.37	0.16	6.01	−1.10*	5.40			
Confusion-bewilderment (CB)									
Placebo	−0.10	4.80	0.82	6.16	0.00	4.54	0.885	0.956	0.367
CLE	−0.64	4.37	0.71	5.93	−0.97	4.49			
Depression-dejection (DD)									
Placebo	0.90	4.42	0.53	4.86	0.35	5.37	0.312	0.287	0.285
CLE	−0.64	4.37	0.71	5.93	−0.97	4.49			
Fatigue-inertia (FI)									
Placebo	−0.03	4.81	−0.70	4.87	−0.25	5.23 6.82	0.900	0.433	0.294
CLE	−0.05	6.73	0.11	7.05	−1.46				
Tension-anxiety (TA)									
Placebo	−1.70	7.77	−2.58	6.00	−1.48	6.99	0.331	0.469	0.108
CLE	0.10	7.36	−0.18	7.00	−1.90	6.69			
Vigor-activity (VA)									
Placebo	1.58	7.19	1.08	8.23	2.10	8.74	0.141	0.184	0.841
CLE	−0.23	5.94	−1.55	6.20	0.13	7.81			
Friendliness (F)									
Placebo	0.05	8.19	−0.63	9.62	− 0.33	8.46	0.983	0.706	0.989
CLE	0.00	7.19	−0.45	7.83	−0.15	8.79			
Total mood disturbance (TMD)									
Placebo	−0.05	4.62	−0.65	4.15	−0.28	3.89	0.894	0.374	0.109
CLE	−0.23	5.04	0.68	6.54	−1.08	5.48			

^a^Values represent the means and standard deviations for n = 40 (placebo group) or n = 39 (CLE group). *p < 0.05: Significant difference from the placebo group by repeated measures two-way ANOVA, followed by the two-tailed unpaired Student’s *t* test when variance was homogeneous or the Aspin-Welch *t* test when variance was heterogeneous

### Safety of the intervention

Adverse events were assessed in the ITT population (placebo group, *n* = 45; CLE group, n = 45). In the placebo group, the following adverse events occurred: headache (three cases), headache and abdominal pain (one case), headache and mood disturbance (one case), pharyngeal pain (one case), chest pain (one case), abdominal pain (one case), common cold symptoms (seven cases), diarrhea (three cases), stomach upset (two cases), dental decay (two cases), fatigue (one case), insect bite (one case), hives (one case), foot blister (one case), scratch (one case), abdominal fullness (one case), positive urinary blood (one case), increased CRP (four cases), increased γ-glutamyl transferase (two cases), increased creatinine phosphokinase (two cases), increased aspartate aminotransferase (two cases), increased body weight (two cases), and decreased body weight (one case); and in the CLE group, the following adverse events occurred: headache (one case), lower back pain (two cases), stomach pain (one case), knee pain (one case), arthritis pain (one case), calf cramp (one case), stiff joint (one case), common cold symptoms (eleven cases), cough (four cases), nasal discharge (two cases), bone fracture (two cases), fatigue (one case), uveitis (one case), insect bite (one case), scratch (one case), suppuration (one case), positive urinary protein (two cases), increased CRP (nine cases), increased creatinine phosphokinase (five cases), increased γ-glutamyl transferase (four cases), increased alanine aminotransferase (one case), increased total bilirubin (one case), increased body weight (one case), and decreased body weight (one case). These adverse events were mild, and an experienced physician judged that they were unrelated to the dietary intervention. Safety parameters (hematology and biochemistry tests, urinalysis, and physical measurements and tests) were assessed in the FAS population (placebo group, *n* = 44; CLE group, n = 44) and did not differ significantly between the two groups, except for urinary protein, which increased in four participants in the placebo group but in no participants in the CLE group. Before the study, we conducted an open-label, one-arm safety study in which twenty participants (mean [SD] age 40.0 [13.1] years) consumed excess dose of CLE (10 capsules/day, ie, a 5-fold higher dose than in this study) for 4 weeks; this open-label study found no adverse effects of the intervention on hematology or biochemistry tests, urinalysis, or physical measurements and tests (data not shown).

## Discussion

This 12-week, randomized, double-blind, placebo-controlled study was performed to investigate the effect of a mixture of a hot water extract and a supercritical carbon dioxide extract of *C. longa* (CLE) on chronic inflammation and general health in middle-aged to elderly participants with overweight. We found that intake of CLE significantly improved body weight, BMI, and serum CRP and C3 levels. In addition, the CLE group had a significantly higher MH score on the SF-36 and a significantly lower AH score on the POMS. These results suggest that daily intake of CLE could potentially reduce chronic inflammation and improve mental health and negative mood states.

According to the World Health Organization, overweight and obesity are defined as an abnormal or excessive fat accumulation that presents a risk to health. BMI is the most commonly used indicator of overweight and obesity. BMI and associated factors show seasonal variation; for example, BMI, fat mass, and dietary fat intake were higher and levels of physical activity were lower in winter than in summer [44, 45]. In the present study, we also demonstrated that body weight and BMI increased from baseline (summer) to week 12 (winter) (Table 4). High BMI values are risk factors for metabolic diseases, cardiovascular disease, Alzheimer disease, depression, and cancer [46]. In the present study, the CLE group showed a significantly lower in BMI from baseline at 4, 8, and 12 weeks compared with the placebo group (Table. 4), but the number of participants who reported exercising was not different between the CLE and placebo groups. Animals studies also showed that dietary intake of *C. longa* extract significantly inhibited body weight gain and visceral fat accumulation in high fat diet-induced obese rats [47, 48]. Therefore, CLE may inhibit the accumulation of visceral fat, resulting in improved BMI values. However, future research will need to clarify the effect of CLE on visceral fat volume in humans with computed tomographic scan analysis [49] or abdominal bioelectrical impedance analysis [50].

Acute phase proteins such as CRP and C3 are generally used as systemic inflammatory markers [14, 20]. These proteins have also been reported to show seasonal variation, with higher values in winter than in summer [51–53]. In the present study, we also observed that serum CRP and C3 levels were higher at baseline (summer) than at week 12 (winter) (Table 5). Slight elevations of CRP accurately detected by hsCRP assay are now considered as a marker of low-grade inflammation [3, 4]. Slightly elevated hsCRP (0.07-0.11 mg/dL) was reported to be associated with an increased risk of metabolic syndrome [7], cardiovascular disease [8], coronary heart disease [54], myocardial infarction [55], diabetes [56], and colon cancer [11]. Increases in serum C3 have been related to the development of diseases associated with low-grade inflammation [20], and the disease risks may be reduced by anti-inflammatory agents [57–59]. In the present study in middle-aged and elderly participants with overweight, CLE significantly improved the serum levels of CRP and C3 (Table 5). These results suggest that CLE may reduce the systemic low-grade inflammation associated with aging and obesity and thus may be able to decrease the risk of chronic inflammatory diseases in these populations.

Low-grade inflammation is induced by several factors, including excessive intake of nutrients (fatty acids and glucose) [6], endoplasmic reticulum stress [3], and damage-associated molecular patterns [4]. These triggers have been reported to promote the expression of pro-inflammatory cytokines that induce the production of inflammatory markers, including CRP, complement proteins, and fibrinogen [3, 4, 6]. In the present study, CLE significantly improved CRP and C3 levels but not C4 and fibrinogen levels (Table 5). Activation of transcription factor nuclear factor kappa B (NF-kB) has been known to increase hepatic production of CRP and C3 [14], but the genes of C4 and fibrinogen have no NF-kB binding sites near the promotor region [60–63]. In fact, activators of NF-kB, such as tumor necrosis factor-α (TNF-α) and interleukin-1β (IL-1β), were reported to be unable to induce the production of C4 and fibrinogen [64–66]. A hot water extract of *C. longa* was reported to inhibit TNF-α–induced phosphorylation of I kappa B-alpha (IkBα), which can lead to activation of NF-kB [37]. Furthermore, turmeronol A and turmeronol B isolated from *C. longa* were shown to inhibit lipopolysaccharide-induced NF-kB activation in macrophages [67]. Taken together, these findings suggest that CLE may reduce CRP and C3 but not C4 and fibrinogen through inhibition of the NF-kB pathway.

QOL is defined as an individual’s perception of their position in life embedded in a cultural, social, and environmental context [68]. Health-related QOL (HR-QOL) can be evaluated by the self-reported SF-36 questionnaire, which measures physical and mental health. In the present study, CLE significantly improved the SF-36 subscale score for mental health (MH) (Table 6). Chronic low-grade inflammation may be related to impaired HR-QOL [69]: Inflammation is known to reduce cellular energy levels and to cause central nervous system inflammation, circadian dysfunction, and fatigue, which together can lead to mental health problems, such as depression, anxiety, and sleep disturbance [10, 70, 71]. Middle-aged participants with fatigue were reported to have increased plasma CRP levels [72]. A follow-up study showed that high levels of CRP predicted fatigue [73]. In addition, compared with participants without fatigue, participants with fatigue and low-grade inflammation as measured by CRP were shown to have mental health problems, including psychological distress, depression, and sleep problems [72]. Previously, etiological studies showed a negative association between serum CRP levels and the SF-36 MH score in patients with a neural injury, such as spinal cord injury [21]. Depressed participants were reported to have significantly higher serum C3 levels than healthy participants [74]. In previous clinical studies, daily intake of medicinal foods with anti-inflammatory activity improved the SF-36 MH score in healthy individuals with normal weight and overweight [75, 76]. Therefore, CLE may improve mental health by alleviating undesirable symptoms related to inflammation.

The POMS is a widely used self-reported questionnaire that measures negative and positive mood states, and it also has been used to evaluate mood disturbance [41]. In the present study, intake of CLE significantly improved negative mood, as indicated by improvement in the POMS AH score (Table 7). Negative mood states are known to be influenced by inflammation. Cross-sectional studies found a significant positive correlation between anger and hostility and serum CRP levels [22]. A previous clinical study showed that a social stress task, the Trier Social Stress Test, not only increased systemic levels of CRP [77] and inflammatory cytokines, but also caused mood disturbance, including anger [78] and anxiety [79]. In a follow-up study, participants with psychological problems (anger and hostility) were shown to have higher levels of serum C3 than healthy participants [23]. Intervention studies demonstrated that daily intake of the medical herb Hochuekkito, which has anti-inflammatory activity [80], improves the AH POMS score in elderly subjects [81]. Therefore, in our study CLE may have improved the negative mood state of anger and hostility by reducing systemic low-grade inflammation.

Mental health problems and mood disturbances are known to be associated with not only systemic inflammation [79, 82] but also neuroinflammation [83, 84]. Brain macrophages, also known as microglial cells, are central players in promoting the development of neuroinflammation by producing inflammatory cytokines that lead to the production of indoleamine 2, 3-dioxygenase (IDO) [83]. Induction of IDO expression promotes the metabolism of tryptophan into the neurotoxic metabolites of kynurenine and decreases the level of natural antidepressant compounds such as serotonin, which is derived from tryptophan [82]. Depression-like symptoms were shown to improve in animal models treated with IDO inhibitors [84]. In addition, a systematic review and meta-analysis of 18 clinical trials found an antidepressant effect for minocycline, a suppressor of microglial activation [85]. In activated microglial cells, *C. longa* extract, turmeronol A, and turmeronol B were found to inhibit the production of inflammatory mediators, including TNF-α and IL-6 [86, 87]. In addition, in animal studies *C. longa* extract inhibited the neuroinflammation associated with fatigue [88], depression [27], memory impairment [86], and anxiety- and sleep deprivation-induced behavior abnormalities [89, 90]. In the present study, dietary supplementation with CLE improved mental health and negative mood state, suggesting that these improvements could be related in part to inhibition of microglial activation in the central nervous system. Future studies need to investigate the effect of CLE on human microglial activation.

## Conclusion

We conducted a 12-week, randomized, double-blind, placebo-controlled study in middle-aged and elderly participants with overweight. Compared with the placebo group, body weight, BMI, and serum levels of CRP and C3 were significantly lower in the CLE group. In addition, the CLE group showed a significant improvement in the SF-36 subscale score for mental health and the POMS score for anger and hostility. These results suggest that intake of a mixture of a hot water extract and supercritical carbon dioxide extract of *C. longa* may have the potential to improve mental health and negative mood state by reducing chronic low-grade inflammation.

## Supplementary Information

**Additional file 1: Table S1**. CONSORT 2010 checklist of information to include when reporting a randomized trial^*^.

## Data Availability

Not applicable.

## Abbreviations

AH: Anger-hostility
ANOVA: Analysis of variance
BMI: Body mass index
CRP: C-reactive protein
CLE: A mixture of a hot water extract and a supercritical carbon dioxide extract of *Curcuma longa*
C3: Complement component 3
C4: Complement component 4
FAS: Full analysis set
hsCRP: High-sensitive C-reactive protein
HR-QOL: Health-related QOL
IL: Interleukin
ITT: Intension-to-treat
MH: Mental health
NF-kB: Nuclear factor kappa B
POMS: Profile of mode state
PPS: Per-protocol set
QOL: Quality of life
r-ANOVA: Repeated measures two-way ANOVA
MOS SF-36: Medical Outcomes Study 36-item short-form health survey
TNF-α: Tumor necrosis factor-alpha
UMIN: University hospital Medical Information Network
